# Disutility associated with social isolation and loneliness in Germany: results of a population survey using the EQ-5D-5L instrument

**DOI:** 10.1186/s12955-024-02329-9

**Published:** 2024-12-20

**Authors:** Hans-Helmut König, André Hajek

**Affiliations:** Department of Health Economics and Health Services Research, Hamburg Center for Health Economics, University Medical Center Hamburg-Eppendorf, Martinistraße 52, 20246 Hamburg, Germany

**Keywords:** EQ-5D, Health-related quality of life, Health state utilities, Disutility, Loneliness, Social isolation

## Abstract

**Background:**

Social isolation and loneliness are highly prevalent and may have a negative impact on health-related quality of life (HRQL). The EQ-5D-5L is a widely used questionnaire from which an index value for HRQL based on societal preferences (utility) can be derived. The purpose of this study was to estimate the loss in utility (i.e. disutility) associated with loneliness and social isolation in the German adult population.

**Methods:**

Data came from a quota sample of individuals aged 18–74 years residing in Germany (*n* = 5,000) and representing the adult population in terms of age groups, gender and geographic locations. Data collection was conducted online in August and September 2023 by a certified market research firm. The EQ-5D-5L index score based on the German value set was used as outcome. Moreover, the established Lubben Social Network Scale was used to measure social isolation. The De Jong Gierveld tool was used to quantify loneliness. Groups affected by social isolation or loneliness were compared to non-affected groups, respectively. Differences in covariates between affected and non-affected groups were balanced using entropy balancing. Linear regressions were conducted afterwards (using the weights derived from the entropy balancing). Subgroup analyses by sex and age groups as well as various robustness checks were conducted.

**Results:**

The EQ-5D-5L index score was lower among individuals with social isolation compared to individuals without social isolation (β=-0.04, *p* < 0.001). Moreover, the EQ-5D-5L index score was lower among individuals with loneliness compared to individuals without loneliness (β=-0.07, *p* < 0.001). Several robustness checks produced similar results. The findings were almost the same for both women and men and varied only slightly between age groups.

**Conclusions:**

We found a statistically significant and relevant disutility associated with social isolation and, even more pronounced, with loneliness. The magnitude of disutilities is similar to those reported for various chronic diseases. Taking into account the high prevalence of social isolation and, in particular, loneliness, the associated burden in terms of quality-adjusted life years (QALY) lost is likely to be tremendous. The results underline the need to take action against the high prevalence of loneliness and social isolation.

**Supplementary Information:**

The online version contains supplementary material available at 10.1186/s12955-024-02329-9.

## Introduction

Social isolation and loneliness are related but distinct concepts. Social isolation refers to individuals being separated from others and the society, and encompasses the quantity and quality of social ties a person maintains [1]. Loneliness refers to “the unpleasant experience that occurs when a person’s network of social relations is deficient in some important way, either quantitatively or qualitatively” [2]. Thus, while social isolation can be considered an objective state, loneliness is a subjective experience. Although both concepts are correlated, they are not identical as a person can be socially isolated without feeling lonely, and vice versa.

Loneliness and social isolation are considered major public health problems, as they are highly prevalent and associated with adverse health outcomes in terms of morbidity [3] and mortality [4]. Furthermore, several studies have shown a negative association of loneliness and/or social isolation with health related quality of life (HRQL) in various general population groups [5–9] and patient samples [10; 11]. In health economic research, HRQL is commonly quantified by so-called (health state) utility values. A utility value is a preference-based index value assigned to a certain state of HRQL on a scale where 0 equals death and 1 is considered perfect health. Utility values are commonly used to calculate quality-adjusted life years (QALY) in cost-utility analyses.

The most frequently used instrument to derive utility values is the EQ-5D quality of life questionnaire [www.euroqol.org; [12]]. The EQ-5D provides a profile of HRQL which can be converted into a utility value (EQ-5D index) based on country-specific value sets representing societal preferences. The underlying algorithm reflects the relative importance of problems in various domains of HRQL as assessed by the general population. EQ-5D value sets and population norms are available for numerous countries including Germany. Utility values can be used to compare HRQL across various diseases and conditions. Furthermore, by comparing utility values of individuals affected by a certain condition with those of an unaffected but otherwise similar control group, the loss in utility attributable to the condition (so-called disutility) can be estimated. Disutility values can be used in model-based economic evaluations or for estimating the burden of a condition in terms of QALYs lost.

According to a recent systematic literature review [13], only few studies have analyzed utility values associated with loneliness and social isolation. All of these studies focused on specific populations *with preexisting health conditions*. While the reviewed studies generally showed a negative association between loneliness/social isolation and utility, there was a great variation in reported utility values, which the authors attributed, amongst others, to comorbidities, population heterogeneity, and differences in methods used to derive utility values and measuring loneliness and social isolation.

Against the background that studies on the loss of utility associated with loneliness and social isolation *in the general population* are missing, the aim of the present study was to analyze the disutility associated with social isolation and loneliness in the German general adult population based on the EQ-5D index and valid, widely used measures for social isolation and loneliness. Thereby this study aims to provide first insights into the disutility burden associated with social isolation and loneliness in the general population as well as subgroups defined by age and sex.

## Methods

### Sample

Data was extracted from a sample of 5,000 individuals residing in Germany, ranging in age from 18 to 74 years. The data collection process took place in the months of August and September 2023. Regarding participant recruitment, the market research firm Bilendi (ISO certified, 26362) was responsible for this task. Participants were selected from an online pool in accordance with specific quotas designed to ensure representation across age groups, genders, and geographic locations, mirroring the demographic composition of the broader adult population in Germany. Data collection was conducted via online questionnaires.

Prior to their involvement in the study, each participant granted their informed consent. Furthermore, the study received official approval from the Local Psychological Ethics Committee at the University Medical Center Hamburg-Eppendorf (LPEK-0629).

### Outcome: EQ-5D-5L index

The EQ-5D-5L questionnaire comprises five items that address current issues in the following domains of HRQL: ‘mobility’, ‘self-care’, ‘usual activities’, ‘pain/discomfort’, and ‘anxiety/depression’ [www.euroqol.org]. Within each of these domains, the 5L-version elicits responses on a five-level ordinal scale, coded as (1) no problems, (2) slight problems, (3) moderate problems, (4) severe problems and (5) extreme problems/unable. This part of the questionnaire is called EQ-5D descriptive system and provides a profile of HRQL represented by five-digit numerical code. For instance, the code ‘11132’ signifies slight problems in anxiety/depression and moderate problems in pain/discomfort, but no problems in the other three dimensions.

The HRQL profile provided by the EQ-5D-5L descriptive system can be converted into a utility value (EQ-5D-5L index) based on country-specific value sets which assign a utility score to each of the 3,125 possible EQ-5D-5L health states, with the best state (no problems in any EQ-5D dimension) and death being assigned values of 1 and 0, respectively. These value sets originate from surveys conducted in various countries, including a representative (quota-based sampling: sex, age, education and employment status) sample from the German general population (*n* = 1,158) [14]. In the aforementioned study, the value set was based on a hybrid model (composite time trade-off and discrete choice experiment). The EQ-5D-5L-index values of the German version can range from − 0.661 to 1. This value set was used in our study for calculating the EQ-5D-5L index.

It is worth noting that the EQ-5D-5L also includes a visual analogue scale (known as EQ-VAS). The EQ-VAS records self-rated health on a scale ranging from 0 (indicating the worst imaginable health) to 100 (indicating the best imaginable health). In contrast to the EQ-5D index, the EQ-VAS score is based on the respondent’s preferences rather than general population preferences. Furthermore, elicitation of the EQ-VAS score is not choice-based and resulting scores have been criticized for not being an interval scale of preferences [15]. Thus, we report EQ-VAS scores for descriptive purposes only.

We decided to use the five level version of the EQ-5D (EQ-5D-5L) because it has been shown to be superior to the three level version (EQ-5D-3L) in terms of ceiling effects, convergent validity and sensitivity to change [16–18].

### Key independent variables: social isolation and loneliness

The 6-item version of the Lubben Social Network Scale (abbreviated as LSNS-6) [19] was employed to assess levels of social isolation in our study. The total score on this scale can vary from 0 to 30, with higher scores indicating lower levels of social isolation. In accordance with established guidelines, individuals with a score below 12 were categorized as ‘socially isolated’, while those with higher scores were categorized as ‘not socially isolated’ [19]. Our study yielded a Cronbach’s alpha reliability coefficient of 0.87 (McDonald’s omega was 0.85). Previous research has also reported favorable psychometric properties for this scale [19].

The assessment of loneliness utilized the 6-item version of the De Jong Gierveld Loneliness scale, with three items subjected to recoding [20]. The final score ranges from 0 to 6, where higher scores indicate greater loneliness. Following the recent recommendations of van Tilburg and De Jong Gierveld [21], individuals scoring between 0 and 1 were categorized as ‘not lonely’, while those with higher scores were classified as ‘lonely’ in our current study. Our study yielded a Cronbach’s alpha coefficient of 0.80 (McDonald’s omega was 0.78), affirming the scale’s favorable psychometric properties [20; 22]. It is worth noting that the tools used in the current study are widely used to quantify loneliness or social isolation in Germany and also worldwide [23–26].

### Statistics

Groups affected by social isolation or loneliness were compared to non-affected groups (control groups), respectively. We applied an entropy balancing (EB) technique to adjust for group differences. In contrast to other common preprocessing methods like propensity score matching, this reweighting strategy demonstrates superior covariate balancing [27]. Specifically, each observation in the control group received a weight from the EB process, aiming to align it with the treatment group [27]. Additionally, former research concluded that “EB is a very appealing alternative to the conventional weighting estimators” [28]. Similarly, another simulation study concluded that “entropy balancing merits more widespread adoption in applied studies.” (p. 491) [29]. In this study, the control group was matched for sex (men; women; diverse), age (in years), education (CASMIN [30]: low education (e.g., basic vocational qualification); medium education (e.g., intermediate vocational qualification); high education (e.g., tertiary education); for further details, please see: [30]), marital status (single; divorced; widowed; living together: married/partnership; living separated: married/partnership), employment status (full-time employed; retired; other), having a migration background (no; yes), and chronic conditions (count score based on the presence of fourteen chronic conditions (in each case: 0 = absence; 1 = presence): Sleep disorder; Thyroid disease; Diabetes; Asthma; Heart disease (also heart failure, cardiac insufficiency); Cancer; Stroke; Migraine; High blood pressure; Dementia; Joint disease (also arthrosis, rheumatism); Chronic back problems; Burnout; Other illness). Of note, individuals were considered to have a migration background if they or at least one of their parents was born without German citizenship – which is a widely accepted way for quantifying a migration background.

Following the EB procedure, we conducted regression analyses to investigate the relationship between isolation/loneliness and HRQL. These regression analyses were weighted using the weights derived from the EB approach. Consequently, only the variable referring to isolation/loneliness was used as the explanatory variable in the subsequent regression analysis.

First, we used simple logistic regression to analyze the association between isolation/loneliness and frequency of problems in the five EQ-5D-5L dimensions. Due to skewness of data and for simplification, the five items were dichotomized (0 = no problems in the respective dimension; 1 = problems in the respective dimension (including slight problems, moderate problems, severe problems and extreme problems).

Afterwards, we conducted simple linear regression to investigate the association between isolation/loneliness and EQ-5D index scores. We conducted subgroup analyses of women and men as well as three age groups (18 to 39 years; 40 to 59 years; 60 to 74 years). Please note that EB was conducted separately for each subsample using all variables mentioned above except for the respective group variable. Robustness checks used coarsened exact matching (CEM) or inverse probability weighting (IPW) instead of EB prior to regression analysis. In the case of coarsened exact matching, the “cem” [31] command was used (for inverse-probability weighting, the Stata command “teffects ipw” was used). The first method (CEM) conducts exact matching on coarsened data to identify matches. It then passes the uncoarsened data from the matched observations to estimate the effect (for further details, please see: [31]). IPW estimators use a two-step approach for estimating the treatment effects. First, the parameters of the treatment model are estimated and the estimated inverse-probability weights are computed. Then, the estimated inverse-probability weights are used to compute weighted averages of the outcomes (for each level of treatment; for further details, please see [32]). Moreover, a conventional multiple linear regression was used to further check the robustness of our results.

We set the threshold for statistical significance at *p* < 0.05. These analyses were performed using Stata 16.1 (StataCorp, College Station, TX, USA), and the “ebalance” package was employed for the EB process [27]. The “omegacoef” tool was used to calculate McDonald’s omega [33].

## Results

### Sample characteristics

The mean age of the participants was 46.9 years (standard deviation: 15.3), with ages spanning from 18 to 74 years. 50.8% of the participants identified as female. The composition of the sample by age group, gender and federal state corresponded well to the total German population aged 18 to 74 years (Supplementary Table 1). Mean EQ-5D-5L-index score equaled 0.88 (SD: 0.18), ranging from − 0.58 to 1. Mean social isolation score was 14.4 (SD: 6.1; from 0 to 30), and 29.8% of the participants can be categorized as socially isolated (LSNS-6 score below 12). The mean loneliness score was 3.1 (SD: 2.1; from 0 to 6), and 72.9% can be categorized as lonely (De Jong Gierveld tool greater than 1).

Table 1 offers insights into the sociodemographic profile of the sample, both before and after applying the EB adjustment. Virtually the same distributions were identified following the EB adjustment. For example, with the EB adjustment, individuals without social isolation had an average age of 50.8 years – which is equal to individuals with social isolation. After EB adjustment, the proportions of women were also identical between individuals with social isolation and individuals without social isolation. More details are shown in Table 1. Furthermore, descriptive data on the frequencies of problems in the EQ-5D dimension as well as EQ-VAS and EQ-5D-5L index scores (stratified by loneliness and social isolation) are shown in Supplementary Table 2.

**Table 1:** Sample characteristics before and after group adjustment by entropy balancing

	Individuals with social isolation	Individuals without social isolation		Individuals with loneliness	Individuals without loneliness	
Variables	*N* = 1491 Mean (SD) / n (%)	Unbalanced (*n* = 3509) Mean (SD) / n (%)	Balanced (*N* = 3509) Mean (SD) / n (%)	*N* = 3643 Mean (SD) / n (%)	Unbalanced *N* = 1357 Mean (SD) / n (%)	Balanced *N* = 1357 Mean (SD) / n (%)
Sex						
Men	733 (49.2%)	1718 (49.0%)	1725 (49.2%)	1797 (49.3%)	654 (48.2%)	669 (49.3%)
Women	756 (50.7%)	1784 (50.8%)	1779 (50.7%)	1839 (50.5%)	701 (51.7%)	685 (50.5%)
Diverse	2 (0.1%)	7 (0.2%)	5 (0.1%)	7 (0.2%)	2 (0.1%)	3 (0.2%)
Age	50.8 (14.2)	45.2 (15.4)	50.8 (14.2)	46.0 (15.2)	49.3 (15.1)	46.0 (15.2)
Marital status						
Single	490 (32.9%)	843 (24.0%)	1153 (32.9%)	1117 (30.7%)	216 (15.9%)	416 (30.7%)
Divorced	193 (12.9%)	210 (6.0%)	454 (12.9%)	315 (8.6%)	88 (6.5%)	117 (8.6%)
Widowed	55 (3.7%)	105 (3.0%)	129 (3.7%)	117 (3.2%)	43 (3.2%)	44 (3.2%)
Living together: Married/Partnership	693 (46.5%)	2200 (62.7%)	1632 (46.5%)	1956 (53.7%)	937 (69.0%)	729 (53.7%)
Living separated: Married/Partnership	60 (4.0%)	151 (4.3%)	141 (4.0%)	138 (3.8%)	73 (5.4%)	51 (3.8%)
Education						
Low	246 (16.5%)	287 (8.2%)	579 (16.5%)	415 (11.4%)	118 (8.7%)	155 (11.4%)
Medium	965 (64.7%)	2022 (57.6%)	2270 (64.7%)	2209 (60.6%)	778 (57.3%)	823 (60.6%)
High	280 (18.8%)	1200 (34.2%)	661 (18.8%)	1019 (28.0%)	461 (34.0%)	379 (28.0%)
Employment status						
Full-time employed	564 (37.8%)	1854 (52.8%)	1328 (37.8%)	1723 (47.3%)	695 (51.2%)	642 (47.3%)
Retired	406 (27.2%)	594 (16.9%)	955 (27.2%)	709 (19.5%)	291 (21.4%)	264 (19.5%)
Others	521 (34.9%)	1061 (30.2%)	1226 (34.9%)	1211 (33.2%)	371 (27.3%)	451 (33.2%)
Migration background						
No	1336 (89.6%)	3113 (88.7%)	3144 (89.6%)	3180 (87.3%)	1269 (93.5%)	1185 (87.3%)
Yes	155 (10.4%)	396 (11.3%)	365 (10.4%)	463 (12.7%)	88 (6.5%)	172 (12.7%)
Count score: chronic conditions	1.9 (1.8)	1.3 (1.5)	1.9 (1.8)	1.6 (1.7)	1.2 (1.4)	1.6 (1.7)

SD, standard deviation

### Main findings

Logistic regression analysis based on the balanced sample showed that individuals with social isolation were more likely to report problems in the dimensions anxiety/depression (OR = 1.68, *p* < 0.001), usual activities (OR = 1.34, *p* < 0.01), mobility (OR = 1.28, *p* < 0.01) and pain/discomfort (OR = 1.24, *p* < 0.01) (Table 2). Individuals with loneliness were more likely to report problems in all EQ-5D dimension: anxiety/depression (OR = 3.78, *p* < 0.001), self-care (OR = 3.51, *p* < 0.001), usual activities (OR = 2.26, *p* < 0.001), pain/discomfort (OR = 1.75, *p* < 0.001) and mobility (OR = 1.69, *p* < 0.01) (Table 3).

**Table 2:** Association of social isolation with problems (0 = no problem; 1 = any problem) in the EQ-5D dimensions^a^

	Mobility OR (95% CI)	Self-care OR (95% CI)	Usual activities OR (95% CI)	Pain/discomfort OR (95% CI)	Anxiety/depression OR (95% CI)
Presence of social isolation (Reference category: absence of social isolation)	1.28 (1.11 to 1.48)**	0.97 (0.77 to 1.21)	1.34 (1.15 to 1.56)**	1.24 (1.07 to 1.43)**	1.68 (1.45 to 1.95)***
Observations	5,000	5,000	5,000	5,000	5,000

*** *p* < 0.001, ** *p* < 0.01, * *p* < 0.05, + *p* < 0.10; OR, odds ratio; CI, confidence interval

^a^ Results of logistic regression analysis; control group matched for age, sex, marital status, employment status, education, migration and chronic conditions using entropy balancing

**Table 3:** Association of loneliness with problems (0 = no problem; 1 = any problem) in the EQ-5D dimensions^a^

	Mobility OR (95% CI)	Self-care OR (95% CI)	Usual activities OR (95% CI)	Pain/discomfort OR (95% CI)	Anxiety/depression OR (95% CI)
Presence of loneliness (Reference category: absence of loneliness)	1.69 (1.53 to 1.87)***	3.51 (2.89 to 4.26)***	2.26 (2.03 to 2.52)***	1.75 (1.60 to 1.92)***	3.78 (3.40 to 4.20)***
Observations	5,000	5,000	5,000	5,000	5,000

*** *p* < 0.001, ** *p* < 0.01, * *p* < 0.05, + *p* < 0.10; OR, odds ratio; CI, confidence interval

^a^ Results of logistic regression analysis; control group matched for age, sex, marital status, employment status, education, migration and chronic conditions using entropy balancing

Linear regression showed that the EQ-5D-5L index score was lower among individuals with social isolation compared to individuals without social isolation (β=-0.04, *p* < 0.001; see Table 4). Moreover, the EQ-5D-5L index score was lower among individuals with loneliness compared to individuals without loneliness (β=-0.07, *p* < 0.001; see Table 5).

**Table 4:** Difference in EQ-5D-5L-Index between individuals with and individuals without social isolation in total sample, and stratified by sex and by age group^a^

	EQ-5D-5L-Index (95% CI)					
	Total sample	Men	Women	18 to 39 years	40 to 59 years	60 to 74 years
Presence of social isolation (Reference category: absence of social isolation)	− 0.04 (-0.05 to − 0.02)***	− 0.04 (-0.06 to − 0.02)***	− 0.04 (-0.07 to − 0.02)***	− 0.02 (-0.04 to 0.002)+	− 0.04 (-0.07 to − 0.01)**	− 0.04 (-0.06 to − 0.02)**
Observations	5,000	2,451	2,540	1,767	2,003	1,230

*** *p* < 0.001, ** *p* < 0.01, * *p* < 0.05, + *p* < 0.10; CI, confidence interval

^a^ Results of simple linear regression; control group matched for age, sex, marital status, employment status, education, migration and chronic conditions using entropy balancing

**Table 5:** Difference in EQ-5D-5L-Index between individuals with and individuals without loneliness in total sample, and stratified by sex and by age group^a^

	EQ-5D-5L-Index (95% CI)					
	Total sample	Men	Women	18 to 39 years	40 to 59 years	60 to 74 years
Presence of loneliness (Reference category: absence of loneliness)	− 0.07 (-0.08 to − 0.05)***	− 0.07 (-0.08 to − 0.05)***	− 0.07 (-0.08 to − 0.05)***	− 0.07 (-0.09 to − 0.05)***	− 0.07 (-0.09 to − 0.04)***	− 0.05 (-0.08 to − 0.03)***
Observations	5,000	2,451	2,540	1,767	2,003	1,230

*** *p* < 0.001, ** *p* < 0.01, * *p* < 0.05, + *p* < 0.10; CI, confidence interval

^a^ Results of simple linear regression; control group matched for age, sex, marital status, employment status, education, migration and chronic conditions using entropy balancing

### Subgroup analyses

We also performed analyses of EQ-5D-5L index scores stratified by sex (men; women) and age group (18 to 39 years; 40 to 59 years; 60 to 74 years) (also presented in Tables 4 and 5). The findings were almost the same (in terms of effect sizes and significance) for both women and men. This applies to both independent variables of interest (social isolation and loneliness).

While the EQ-5D-5L index was reduced in individuals with social isolation in the older age groups 40–59 years and 60 to 74 years (in each case: β=-0.04, *p* < 0.01), there was no statistically significant reduction in EQ-5D-5L index scores in the younger age group 18 to 39 years. For individuals with loneliness, the EQ-5D-5L index was statistically significantly reduced in all age groups.

### Robustness checks

As described in the statistical analysis section, we conducted robustness checks on the association between EQ-5D-5L index and social isolation as well as loneliness. For social isolation (see Supplementary Table 3): When using a coarsened exact matching approach prior to regression analysis, results remained very similar (β=-0.03, *p* < 0.001). Similarly, when inverse probability weighting was used prior to regression analysis, the coefficient was virtually the same compared to our main model (β=-0.04, *p* < 0.001). Moreover, when we used a conventional multiple linear regression (adjusted for sex, age, employment status, education, migration and chronic conditions), the findings remained very similar (β=-0.04, *p* < 0.001).

For loneliness (see Supplementary Table 4): Based on a coarsened exact matching approach prior to regression analysis, the beta-coefficient was β=-0.04 (*p* < 0.001). With an inverse probability weighting approach, the results were very similar (β=-0.06, *p* < 0.001) compared to our main approach. Furthermore, with a conventional multiple linear regression approach (again: adjusted for sex, age, employment status, education, migration and chronic conditions), the findings remained similar (β=-0.05, *p* < 0.001).

Corresponding robustness checks stratified by sex yielded stable results, and can be found for loneliness in Supplementary Tables 5 and for social isolation in Supplementary Table 6.

While the significance for the association between presence of social isolation and EQ-5D-5L index among individuals aged 18 to 39 years depended on the analytical approach used, there were statistically significant associations between these variables in the other age groups (except for the model with coarsened exact matching among individuals aged 60 to 74 years) (Supplementary Table 7). Moreover, comparable statistically significant associations were identified between the presence of loneliness and EQ-5D-5L index among the three age groups (Supplementary Table 8).

## Discussion

We found statistically significant disutilities associated with social isolation and, even more pronounced, with loneliness. Not surprisingly, the disutility associated with loneliness was greater than for social isolation, as loneliness is an unpleasant subjective experience whereas rather objective social isolation does not need not be considered unpleasant by the affected person and thus may not reduce HRQL. The magnitude of disutilities may be considered (clinically) relevant as they tended to be similar to the minimally important difference for EQ-5D index scores (generally estimated between 0.04 and 0.07 [34]) and similar to disutilities reported for various chronic diseases: According to a recent systematic review of disutilies based on EQ-5D index scores, the disutility of 0.04 found for social isolation is similar to disutilities reported, e.g., for type 2 diabetes or osteoporosis by several studies, whereas the disutility of 0.07 associated with loneliness is similar to, e.g., disutilities reported for various types of visual impairment or back pain [35]. Taking into account prevalence rates of 29.8% and 72.9%, respectively, as well as approximately 61.5 million people being aged 18 to 74 years in Germany [36], the disutilities found would translate into approximately 734 thousand QALY lost associated with social isolation and even 3.14 million QALY lost associated with loneliness in 2023 in the considered age group alone. Thus, the results underline the need to take action against the high prevalence of loneliness and social isolation.

The disutility associated with social isolation and loneliness varied only little by sex and age group, with the exception of no statistically significant disutility associated with social isolation in the youngest age group 18–29 years. This may be due to the higher preference for social media use among younger people [37]. It has been suggested that people who prefer social media also desire activities that are more isolated in nature [38]. Other potential explanations could be related to the current multiple crises (e.g., new emerging wars, climate change and Covid-19 pandemic) that affect young adults in the formative stages of their lives (school leaving, university studies, first job) [39–41]. It is worth noting that the disutility associated with loneliness was similar in the younger age group compared to the total sample.

Both, social isolation and loneliness were most strongly associated with problems in the EQ-5D dimensions anxiety/depression, but associations were also statistically significant with all other EQ-5D dimension (except for self-care with social isolation). All association of problems in EQ-5D dimension were stronger with loneliness than social isolation.

One has to keep in mind that the prevalence rates of loneliness and social isolation depend on the respective measurement scales and their cutoff scores. We used psychometrically sound and well-established scales, and cutoff scores recommended by their developers. The prevalence of loneliness and social isolation found in our study is very similar to the results of a German general population online survey (*N* = 3075; age range: 18 to 70 years) conducted in 2021 which used the same measurement scales and cutoff scores (prevalence of loneliness: 83.4%; of social isolation 28.9%) [42]. Yet, a general adult population survey conducted in Leipzig, Germany (*N* = 9392, age range: 18–79) found a comparatively lower prevalence of social isolation of only 12.3% based on the same scale (LSNS-6) and cutoff score [43]. This difference might be due to the latter study having been conducted before the pandemic (2011–2014), in a single city and by using personal interviews. Other surveys which assessed loneliness in the German general adult population in 2018 and 2020 [44] found markedly lower prevalence rates of only 23–25%. However, the latter surveys used only a single item scale which is difficult to compare to the validated multi-item scale (De Jong Gierveld Loneliness scale) used in our study. Of note, when we use a threshold of values above three, mirroring the midpoint of the scale [45], to indicate loneliness (which results in a prevalence of 44.3% for loneliness), the association between the presence of loneliness and the EQ-5D-5L index score remained nearly the same among the total sample (β=-0.06, 95% CI: − 0.08 to − 0.05, *p* < 0.001) compared to our main model presented in Table 5 (second column).

Notably, certain strengths and weaknesses deserve acknowledgment. This study provides first insights into the disutility associated with social isolation and loneliness in a general population sample. The outcome was assessed with a standard and most frequently used instrument for deriving utility values. The key independent variables were assessed using psychometrically valid tools. Furthermore, advanced tools were used for data analysis. Our results proved to be statistically robust in various robustness checks. However, our investigation was limited to individuals aged 18 to 74. Consequently, there is an imperative need for forthcoming research to encompass individuals aged 75 and beyond. Data were collected via an online panel, which is a cost-effective and speedy way of data collection but might be prone to bias due to self-selection and the fact that not all potential respondents have equal access to the internet. Moreover, the survey was conducted during vacation time in August and September, which might have biased participation. However, since the survey was conducted online, participation was also possible when on vacation. Furthermore, it has to be pointed out that the associations found in our study are based on cross-sectional data which makes causal inference difficult. While loneliness and social isolation might cause a decrease in HRQL, causation could well be the other way round, i.e. low HRQL causing loneliness and social isolation.

In conclusion, our study shows that the disutility associated with social isolation and, in particular, loneliness is substantial. The results underline the need to take action against the high prevalence of loneliness and social isolation.

## Electronic Supplementary Material

Below is the link to the electronic supplementary material.

Supplementary Material 1

## Data Availability

The data cannot be shared publicly because we have no permission from the respondents of our survey to share the de-identified dataset with the general public. Data requests can be directed to the corresponding author (h.koenig@uke.de).
