# Supplementary material for: Disutility associated with social isolation and loneliness in Germany: results of a population survey using the EQ-5D-5L instrument
Source: Health Qual Life Outcomes. 2024 Dec 20;22:110. doi: 10.1186/s12955-024-02329-9 (PMC11662566; doi:10.1186/s12955-024-02329-9)
Supplement: Supplementary file 1 — Supplementary Material 1 [file 12955_2024_2329_MOESM1_ESM.docx]

Supplementary Tables

Supplementary Table 1: Quotas from EUROSTAT 2021 and our sample

|  | Quotas from EUROSTAT 2021 | Our sample |
| --- | --- | --- |
| *Gender* |  |  |
| Female | 50% | 50.8% |
| Male | 50% | 49.0% |
| Diverse |  | 0.2% |
| *Age* |  |  |
| 18 - 29 years | 18% | 18% |
| 30 - 39 years | 18% | 18% |
| 40 - 49 years | 17% | 17% |
| 50 - 59 years | 22% | 23% |
| 60 - 74 years | 24% | 25% |
| *Region* |  |  |
| Baden-Wuerttemberg | 13% | 13% |
| Bavaria | 16% | 16% |
| Berlin | 4% | 4% |
| Brandenburg | 3% | 3% |
| Bremen | 1% | 1% |
| Hamburg | 2% | 2% |
| Hesse | 8% | 8% |
| Mecklenburg-Western Pomerania | 2% | 2% |
| Lower Saxony | 10% | 10% |
| North Rhine-Westphalia | 22% | 21% |
| Rhineland-Palatinate | 5% | 5% |
| Saarland | 1% | 1% |
| Saxony | 5% | 5% |
| Saxony-Anhalt | 3% | 3% |
| Schleswig-Holstein | 3% | 3% |
| Thuringia | 3% | 3% |

Supplementary Table 2. Description of EQ-5D-5L scores in total sample and by presence of social isolation or lonelines

| Variables | Individuals with social isolation  n=1491 | Individuals without social isolation (balanced)  n=3509 | Individuals with loneliness  n=3643 | Individuals without loneliness (balanced)  n=1357 | Total sample  n=5000  (before matching) |
| --- | --- | --- | --- | --- | --- |
|  | Mean (SD) / n (%) | Mean (SD) / n (%) | Mean (SD) / n (%) | Mean (SD) / n (%) | Mean (SD) / n (%) |
| Mobility |  |  |  |  |  |
| No problems | 844 (56.6%) | 2196 (62.6%) | 2242 (61.5%) | 991 (73.0%) | 3295 (65.9%) |
| Slight problems | 353 (23.7%) | 760 (21.7%) | 810 (22.2%) | 249 (18.4%) | 1023 (20.5%) |
| Moderate problems | 203 (13.6%) | 383 (10.9%) | 430 (11.8%) | 75 (5.5%) | 495 (9.9%) |
| Severe problems | 83 (5.6%) | 148 (4.2%) | 143 (3.9%) | 38 (2.8%) | 165 (3.3%) |
| Extreme problems | 8 (0.5%) | 22 (0.6%) | 18 (0.5%) | 4 (0.3%) | 22 (0.4%) |
| Self-care |  |  |  |  |  |
| No problems | 1326 (88.9%) | 3111 (88.7%) | 3182 (87.3%) | 1303 (96.0%) | 4494 (89.9%) |
| Slight problems | 95 (6.4%) | 261 (7.5%) | 262 (7.2%) | 40 (3.0%) | 295 (5.9%) |
| Moderate problems | 53 (3.6%) | 81 (2.3%) | 142 (3.9%) | 8 (0.6%) | 149 (3.0%) |
| Severe problems | 9 (0.6%) | 40 (1.1%) | 39 (1.1%) | 3 (0.3%) | 42 (0.8%) |
| Extreme problems | 8 (0.5%) | 15 (0.4%) | 18 (0.5%) | 2 (0.2%) | 20 (0.4%) |
| Usual activities |  |  |  |  |  |
| No problems | 932 (62.5%) | 2425 (69.1%) | 2420 (66.4%) | 1109 (81.8%) | 3583 (71.7%) |
| Slight problems | 338 (22.7%) | 704 (20.1%) | 769 (21.1%) | 174 (12.8%) | 915 (18.3%) |
| Moderate problems | 157 (10.5%) | 278 (7.9%) | 331 (9.1%) | 54 (4.0%) | 364 (7.3%) |
| Severe problems | 51 (3.4%) | 79 (2.3%) | 96 (2.6%) | 19 (1.4%) | 110 (2.2%) |
| Extreme problems | 13 (0.9%) | 23 (0.6%) | 27 (0.7%) | 1 (0.1%) | 28 (0.6%) |
| Pain/discomfort |  |  |  |  |  |
| No problems | 546 (36.6%) | 1461 (41.7%) | 1469 (40.3%) | 736 (54.2%) | 2280 (45.6%) |
| Slight problems | 553 (37.1%) | 1321 (37.7%) | 1397 (38.3%) | 451 (33.3%) | 1813 (36.3%) |
| Moderate problems | 276 (18.5%) | 518 (14.8%) | 558 (15.3%) | 126 (9.3%) | 660 (13.2%) |
| Severe problems | 102 (6.8%) | 179 (5.1%) | 190 (5.2%) | 41 (3.0%) | 215 (4.3%) |
| Extreme problems | 14 (0.9%) | 29 (0.8%) | 29 (0.8%) | 3 (0.2%) | 32 (0.6%) |
| Anxiety/depression |  |  |  |  |  |
| No problems | 769 (51.6%) | 2252 (64.2%) | 1934 (53.1%) | 1100 (81.0%) | 3107 (62.1%) |
| Slight problems | 362 (24.3%) | 769 (21.9%) | 970 (26.6%) | 183 (13.5%) | 1109 (22.2%) |
| Moderate problems | 212 (14.2%) | 352 (10.0%) | 501 (13.8%) | 48 (3.5%) | 532 (10.6%) |
| Severe problems | 119 (8.0%) | 109 (3.1%) | 191 (5.2%) | 24 (1.8%) | 203 (4.1%) |
| Extreme problems | 29 (1.9%) | 26 (0.7%) | 47 (1.3%) | 3 (0.2%) | 49 (1.0%) |
|  |  |  |  |  |  |
| At least slight problems in each dimension |  |  |  |  |  |
| No | 1378 (92.4%) | 3247 (92.5%) | 3330 (91.4%) | 1339 (98.6%) | 4674 (93.5%) |
| Yes | 113 (7.6%) | 262 (7.5%) | 313 (8.6%) | 18 (1.4%) | 326 (6.5%) |
|  |  |  |  |  |  |
| EQ-5D-5L-Index | 0.83 (0.22) | 0.87 (0.19) | 0.86 (0.20) | 0.92 (0.14) | 0.88 (0.18) |
|  |  |  |  |  |  |
| EQ-VAS | 63.7 (23.8) | 70.6 (21.5) | 67.6 (22.0) | 77.8 (18.0) | 71.2 (21.4) |

Supplementary Table 3. Robustness check: Difference in EQ-5D-5L-Index between individuals with and individuals without social isolation in total sample based on various methods to control for confounding

|  | EQ-5D-5L Index  (matching approach: entropy balancing^a^) | EQ-5D-5L Index  (matching approach: coarsened exact matching^a^) | EQ-5D-5L Index  (matching approach: inverse probability weighting (probit)^a^) | EQ-5D-5L Index  (without matching, conventional multiple linear regression^b^) |
| --- | --- | --- | --- | --- |
| Presence of social isolation (Reference category: absence of social isolation) | -.04 (-.05 to -.02)*** | -.03 (-.05 to -.01)*** | -.04 (-.05 to -.02)*** | -.04 (-.05 to -.03)*** |
| Observations | 5,000 | 2,877 | 5,000 | 5,000 |

*** p<0.001, ** p<0.01, * p<0.05, + p<0.10

^a^ Control group matched for: age, sex, marital status, employment status, education, migration and chronic conditions.

^b^ It was adjusted for sex, age, employment status, marital status, education, migration and chronic conditions) in the regression model (without prior matching)

Supplementary Table 4. Robustness check: Difference in EQ-5D-5L-Index between individuals with and individuals without loneliness in total sample based on various methods to control for confounding

|  | EQ-5D-5L Index  (matching approach: entropy balancing^a^) | EQ-5D-5L Index  (matching approach: coarsened exact matching^a^) | EQ-5D-5L Index  (matching approach: inverse probability weighting probit)^a^) | EQ-5D-5L Index  (without matching, conventional multiple linear regression^b^) |
| --- | --- | --- | --- | --- |
| Presence of loneliness (Reference category: absence of loneliness) | -.07 (-.08 to -.05)*** | -.04 (-.05 to -.03)*** | -.06 (-.07 to -.05)*** | -.05 (-.06 to -.05)*** |
| Observations | 5,000 | 2,835 | 5,000 | 5,000 |

*** p<0.001, ** p<0.01, * p<0.05, + p<0.10

^a^ Control group matched for: age, sex, marital status, employment status, education, migration and chronic conditions.

^b^ It was adjusted for sex, age, employment status, marital status, education, migration and chronic conditions) in the regression model (without prior matching)

Supplementary Table 5. Robustness check: Difference in EQ-5D-5L-Index between individuals with and individuals without social isolation stratified by sex based on various methods to control for confounding

|  | EQ-5D-5L Index  (matching approach: entropy balancing^a^) | EQ-5D-5L Index  (matching approach: coarsened exact matching^a^) | EQ-5D-5L Index  (matching approach: inverse probability weighting (probit)^a^) | EQ-5D-5L Index  (without matching, conventional multiple linear regression^b^) | EQ-5D-5L Index  (matching approach: entropy balancing^a^) | EQ-5D-5L Index  (matching approach: coarsened exact matching^a^) | EQ-5D-5L Index  (matching approach: inverse probability weighting (probit)^a^) | EQ-5D-5L Index  (without matching, conventional multiple linear regression^b^) |
| --- | --- | --- | --- | --- | --- | --- | --- | --- |
|  | Men | Men | Men | Men | Women | Women | Women | Women |
| Presence of social isolation (Reference category: absence of social isolation) | -.04 (-.06 to -.02)*** | -.04 (-.07 to -.02)*** | -.03 (-.05 to -.02)*** | -.04 (-.05 to -.02)*** | -.04 (-.07 to -.02)*** | -.03 (-.05 to -.01)** | -.04 (-.05 to -.02)*** | -.04 (-.05 to -.02)*** |
| Observations | 2,451 | 1,690 | 2,451 | 2,451 | 2,540 | 1,429 | 2,540 | 2,540 |

*** p<0.001, ** p<0.01, * p<0.05, + p<0.10

^a^ Control group matched for: age, marital status, employment status, education, migration and chronic conditions.

^b^ It was adjusted for age, employment status, marital status, education, migration and chronic conditions) in the regression model (without prior matching)

Supplementary Table 6. Robustness check: Difference in EQ-5D-5L-Index between individuals with and individuals without loneliness stratified by sex based on various methods to control for confounding

|  | EQ-5D-5L Index  (matching approach: entropy balancing^a^) | EQ-5D-5L Index  (matching approach: coarsened exact matching^a^) | EQ-5D-5L Index  (matching approach: inverse probability weighting (probit)^a^) | EQ-5D-5L Index  (without matching, conventional multiple linear regression^b^) | EQ-5D-5L Index  (matching approach: entropy balancing^a^) | EQ-5D-5L Index  (matching approach: coarsened exact matching^a^) | EQ-5D-5L Index  (matching approach: inverse probability weighting (probit)^a^) | EQ-5D-5L Index  (without matching, conventional multiple linear regression^b^) |
| --- | --- | --- | --- | --- | --- | --- | --- | --- |
|  | Men | Men | Men | Men | Women | Women | Women | Women |
| Presence of loneliness (Reference category: absence of loneliness) | -.07 (-.08 to -.05)*** | -.04 (-.06 to -.03)*** | -.07 (-.08 to -.05)*** | -.05 (-.06 to -.04)*** | -.07 (-.08 to -.05)*** | -.05 (-.06 to -.03)*** | -.06 (-.08 to -.04)*** | -.06 (-.07 to -.04)*** |
| Observations | 2,451 | 1,607 | 2,451 | 2,451 | 2,540 | 1,488 | 2,540 | 2,540 |

*** p<0.001, ** p<0.01, * p<0.05, + p<0.10

^a^ Control group matched for: age, marital status, employment status, education, migration and chronic conditions.

^b^ It was adjusted for age, employment status, marital status, education, migration and chronic conditions) in the regression model (without prior matching)

Supplementary Table 7. Robustness check: Difference in EQ-5D-5L-Index between individuals with and individuals without social isolation stratified by age group based on various methods to control for confounding

|  | EQ-5D-5L Index  (matching approach: entropy balancing^a^) | EQ-5D-5L Index  (matching approach: coarsened exact matching^a^) | EQ-5D-5L Index  (matching approach: inverse probability weighting (probit)^a^) | EQ-5D-5L Index  (without matching, conventional multiple linear regression^b^) | EQ-5D-5L Index  (matching approach: entropy balancing^a^) | EQ-5D-5L Index  (matching approach: coarsened exact matching^a^) | EQ-5D-5L Index  (matching approach: inverse probability weighting (probit)^a^) | EQ-5D-5L Index  (without matching, conventional multiple linear regression^b^) | EQ-5D-5L Index  (matching approach: entropy balancing^a^) | EQ-5D-5L Index  (matching approach: coarsened exact matching^a^) | EQ-5D-5L Index  (matching approach: inverse probability weighting (probit)^a^) | EQ-5D-5L Index  (without matching, conventional multiple linear regression^b^) |
| --- | --- | --- | --- | --- | --- | --- | --- | --- | --- | --- | --- | --- |
|  | 18 to 39 years | 18 to 39 years | 18 to 39 years | 18 to 39 years | 40 to 59 years | 40 to 59 years | 40 to 59 years | 40 to 59 years | 60 to 74 years | 60 to 74 years | 60 to 74 years | 60 to 74 years |
| Presence of social isolation (Reference category: absence of social isolation) | -.02 (-.04 to .002)+ | -.02 (-.05 to .004) | -.02 (-.04 to -.001)* | -.02 (-.04 to -.003)* | -.04 (-.07 to -.01)** | -.04 (-.07 to -.01)** | -.03 (-.05 to -.02)*** | -.04 (-.05 to -.02)*** | -.04 (-.06 to -.02)** | -.02 (-.05 to .01) | -.04 (-.06 to -.02)*** | -.04 (-.06 to -.02)*** |
| Observations | 1,767 | 989 | 1,767 | 1,767 | 2,003 | 812 | 2,003 | 2,003 | 1,230 | 418 | 1,230 | 1,230 |

*** p<0.001, ** p<0.01, * p<0.05, + p<0.10

^a^ Control group matched for: sex, age, marital status, employment status, education, migration and chronic conditions.

^b^ It was adjusted for sex, age, employment status, marital status, education, migration and chronic conditions) in the regression model (without prior matching)

Supplementary Table 8. Robustness check: Difference in EQ-5D-5L-Index between individuals with and individuals without loneliness stratified by age group based on various methods to control for confounding

|  | EQ-5D-5L Index  (matching approach: entropy balancing^a^) | EQ-5D-5L Index  (matching approach: coarsened exact matching^a^) | EQ-5D-5L Index  (matching approach: inverse probability weighting (probit)^a^) | EQ-5D-5L Index  (without matching, conventional multiple linear regression^b^) | EQ-5D-5L Index  (matching approach: entropy balancing^a^) | EQ-5D-5L Index  (matching approach: coarsened exact matching^a^) | EQ-5D-5L Index  (matching approach: inverse probability weighting (probit)^a^) | EQ-5D-5L Index  (without matching, conventional multiple linear regression^b^) | EQ-5D-5L Index  (matching approach: entropy balancing^a^) | EQ-5D-5L Index  (matching approach: coarsened exact matching^a^) | EQ-5D-5L Index  (matching approach: inverse probability weighting (probit)^a^) | EQ-5D-5L Index  (without matching, conventional multiple linear regression^b^) |
| --- | --- | --- | --- | --- | --- | --- | --- | --- | --- | --- | --- | --- |
|  | 18 to 39 years | 18 to 39 years | 18 to 39 years | 18 to 39 years | 40 to 59 years | 40 to 59 years | 40 to 59 years | 40 to 59 years | 60 to 74 years | 60 to 74 years | 60 to 74 years | 60 to 74 years |
| Presence of loneliness (Reference category: absence of loneliness) | -.07 (-.09 to -.05)*** | -.06 (-.07 to -.04)*** | -.07 (-.09 to -.06)*** | -.06 (-.07 to -.04)*** | -.07 (-.09 to -.04)*** | -.04 (-.06 to -.03)*** | -.06 (-.08 to -.04)*** | -.05 (-.07 to -.04)*** | -.05 (-.08 to -.03)*** | -.03 (-.05 to -.002)* | -.05 (-.07 to -.03)*** | -.04 (-.06 to -.02)*** |
| Observations | 1,767 | 983 | 1,767 | 1,767 | 2,003 | 901 | 2,003 | 2,003 | 1,230 | 450 | 1,230 | 1,230 |

*** p<0.001, ** p<0.01, * p<0.05, + p<0.10

^a^ Control group matched for: sex, age, marital status, employment status, education, migration and chronic conditions.

^b^ It was adjusted for sex, age, employment status, marital status, education, migration and chronic conditions) in the regression model (without prior matching)
